# Food insecurity measurement and prevalence estimates during the COVID-19 pandemic in a repeated cross-sectional survey in Mexico

**DOI:** 10.1017/S1368980020004000

**Published:** 2020-10-14

**Authors:** Pablo Gaitán-Rossi, Mireya Vilar-Compte, Graciela Teruel, Rafael Pérez-Escamilla

**Affiliations:** 1 Research Institute for Equitable Development EQUIDE, Universidad Iberoamericana, Prolongación Paseo de la Reforma 880, Lomas de Santa Fé, Mexico City 01219, Mexico; 2 Department of Social and Behavioral Sciences, Yale School of Public Health, New Haven, CT, USA

**Keywords:** COVID-19, ELCSA, Food security, Mexico, Validity

## Abstract

**Objective:**

To validate the telephone modality of the Latin American and Caribbean Food Security Scale (ELCSA) included in three waves of a phone survey to estimate the monthly household food insecurity prevalence during the COVID-19 pandemic in Mexico.

**Design:**

We examined the reliability and internal validity of the ELCSA scale in three repeated waves of cross-sectional surveys with Rasch models. We estimated the monthly prevalence of food insecurity in the general population and in households with and without children and compared them with a national 2018 survey. We tested concurrent validity by testing associations of food insecurity with socio-economic status and anxiety.

**Setting:**

ENCOVID-19 is a monthly telephone cross-sectional survey collecting information on the well-being of Mexican households during the pandemic lockdown. Surveys used probabilistic samples, and we used data from April (*n* 833), May (*n* 850) and June 2020 (*n* 1674).

**Participants:**

Mexicans 18 years or older who had a mobile telephone.

**Results:**

ELCSA had an adequate model fit and food insecurity was associated, within each wave, with more poverty and anxiety. The COVID-19 lockdown was associated with an important reduction in food security, decreasing stepwise from 38·9 % in 2018 to 24·9 % in June 2020 in households with children.

**Conclusions:**

Telephone surveys were a feasible strategy to monitor reductions in food security during the COVID-19 lockdown.

The number of people with severe food insecurity has been rising globally since 2014 and the COVID-19 pandemic will likely reduce food security even further^(1)^. Several socio-economic and health pathways can contribute to such reduction. Even though the association between household income and food security is well established^(2)^, the COVID-19 pandemic constitutes an important external socio-economic shock to households because of its effects in unemployment, poverty and a subsequent reduction in food purchases^(3)^. In the USA, households that allocate a higher income share on food are affected the most by economic shocks^(4)^, and previous studies have found that severe economic crises, such as the one in 2008, reduced food security in Mexican households and had a larger effect among households with lower income^(5)^. The economic shock is affecting more strongly households that were already vulnerable prior to the pandemic. Results from the Understanding Coronavirus in America cohort, a longitudinal survey conducted in Southern California, show that 40·5 % of those living in households earning <US$75 000 annually and who were employed in February 2020 have lost their job during the COVID-19 pandemic. Amongst them, 31 % reported food insecurity and 33 % reported eating less due to financial constraints^(6)^. Likewise, an analysis of five repeated online surveys of the US Supplemental Nutritional Assistance Program recipients documented that household food insecurity and financial debt worsened significantly between April and June 2020, when compared with the corresponding period of time in 2018^(7)^. A similar trend has been observed in middle-income countries. An online cross-sectional survey conducted between 27 March and 1 June 2020, in two favelas in Sao Paulo, Brazil, showed that 47 % of respondents experienced moderate or severe food insecurity; 89 % of them reported uncertainty to access food, 64 % eating less than they should, and 39 % skipping a meal^(8)^.

The current pandemic crisis has been long-lasting, and it is also affecting the food security of households that were not poor prior to the pandemic due to debt, temporary or permanent job loss or catastrophic illness^(8)^. In Vermont, USA, a cross-sectional survey collected from 29 March to 12 April 2020 showed a 33 % increase in household food insecurity, with 35·6 % of food insecure households classified as newly food insecure due to the pandemic and job loss being a main predictor of food insecurity^(9)^. An interrupted time series with a probabilistic sample in Bangladesh found sharp reductions in income, food security and mental health. During the lockdown, moderate food insecurity increased from 5·6 to 36·5 % and severe food insecurity did so from 2·7 to 15·3 %^(10)^.

In addition to its negative economic impacts, the needed social distancing measures are disrupting food systems leading to increased food prices and making it more difficult to access healthy foods^(11)^. A cross-sectional study conducted in the USA and China found shifting food consumption patterns during the lockdown, mostly caused by reduced options at supermarkets, and increases in household food insecurity^(12)^. Moreover, these measures may hinder the access to food assistance programmes, such as school meals^(13)^. Thus, a serious concern is that food consumption patterns during the pandemic can have negative health consequences following a syndemic pattern, including obesity and related diseases, as a result of increased consumption of ultra-processed foods, sugar-sweetened beverages, sedentarism and reduced access to health services^(3)^.

Another consequence of experiencing food insecurity is poor mental health. A global analysis of the effects of food insecurity on mental health status found a consistent dose–response; increasing food insecurity amplified diverse psychosocial stressors that were linked to heightened anxiety^(14)^. A recent meta-analysis identified that food insecurity has a significant effect on the likelihood of being stressed or depressed^(15)^. A US study conducted during the pandemic in a convenience sample found that households experiencing food insecurity were 2·09 and 1·88 times more likely to screen positive for anxiety and depression, respectively, than food secure households^(16)^. Food security in households with children requires particular attention^(17)^ because it has been associated with developmental risk and in low- and middle-income countries with lower vocabulary skills^(18)^. These child development delays can be mediated by depression or anxiety in caregivers^(19)^. The economic crises and the social distancing measures have the potential to exacerbate the link between food insecurity and mental health^(3)^.

The disruption of the COVID-19 pandemic can amplify these adverse outcomes because the required attention may be insufficient in fragile health and mental health care systems in low- and middle-income countries^(20)^. The coexistence of COVID-19, food insecurity and anxiety, amidst poverty and unemployment, can be understood as a complex syndemic^(3)^. Syndemics are the aggregation of two or more health conditions driven by common factors which usually are disproportionately common in impoverished populations where health care is limited^(21)^. The long protracted nature of the COVID-19 pandemic will likely worsen this complex syndemic, increasing the severity of food insecurity^(3)^.

Experienced-based food security scales – such as the Escala Latinoamericana y Caribeña de Seguridad Alimentaria (ELCSA)^(22)^ – have been shown to be cost-effective and valid to assess food insecurity via face-to-face interviews in most countries in Latin America, Africa and Asia^(23)^. The COVID-19 pandemic made it impossible to conduct much needed food insecurity monitoring via face-to-face surveys in Mexico. Hence, a repeated, low-cost remote food insecurity assessment was warranted^(24)^. Telephone surveys were considered as a feasible strategy to address this need as interview mode (in-person *v*. telephone) has been shown before to have small effects on food security prevalence estimates; the adjusted OR of food insecurity comparing in-person with telephone interview in the USA was 1·036 (*P* = 0·088)^(25)^. Moreover, experienced-based food security scales had already been applied by telephone in middle- and high-income countries with at least 80 % telephone coverage^(23)^. To the extent of our knowledge, however, the assessment of food insecurity with an experienced-based food security scale via nationally representative phone surveys during a public health emergency has not been properly validated.

The present study has three objectives. First, to compare the psychometric validity and reliability of ELCSA in a large face-to-face survey conducted prior to the pandemic and three waves of an adapted version collected through a telephone survey. Second, to assess the concurrent validity of the adapted ELCSA scale in the telephone survey. Lastly, to estimate the monthly prevalence of food insecurity in the general population and among households with and without children during the lockdown of the COVID-19 pandemic in Mexico.

As many other countries struck by the COVID-19, Mexico implemented a national lockdown measure from 17 March to 31 May. The Mexican government then shifted to a four-level risk system at the state level. However, during June 2020, most of the country remained at the ‘red’ level, indicating the highest risk, in which only essential businesses could operate and most of the households in the country had to maintain the lockdown.

## Methods

### Data source

ENCOVID-19 is a monthly telephone cross-sectional survey, representative at a national level of individuals 18 years and older who have a mobile phone. This initiative is led by an academic research centre in strong partnership with international agencies and local civil society organisations. A key objective of ENCOVID-19 is to provide information to the media and decision-makers on the well-being of Mexican households during the COVID-19 pandemic. ENCOVID-19 is publicly available and offers timely data to decision-makers and the public at large on the social consequences of the lockdown measures in four main domains: labour, income, mental health and food security. It started in April 2020 and will continue for 12 more months^(26)^. The first ENCOVID-19 survey was collected from 6 April to 14 April (*n* 833), the second wave between 20 May and 25 May (*n* 850) and the third wave between 5 June and 17 June (*n* 1674).

The monthly surveys were collected based on a one-stage probabilistic sample of mobile telephone numbers which are randomly selected from the publicly available National Dialing Plan^(27)^. The number selection uses a single stratified random sampling for the thirty-two Mexican states and is implemented with Random Digit Dialing. As of 3 April 2020, the coverage of mobile phones in Mexico was 96 %^(27)^. Previous surveys from the National Institute of Statistics (INEGI for its acronym in Spanish) confirm the wide coverage of mobile phones in Mexico; in 2019, a national survey on the availability and use of technology found the coverage to be 89·4 %, but drops to 74 % in rural areas^(28)^. Since the ENCOVID-19 might not reach isolated communities, post-stratification sampling weights were used to correct for minor deviations from the Mexican population’s demographic structure. Weights were calculated using the 2015 census data from INEGI and adjust the sample by geographic distribution (state) and by sex, age and socio-economic status (SES). Further details of ENCOVID-19 and the composition of the sample are available elsewhere^(26)^.

The monthly surveys were collected by trained interviewers. In addition, a supervisor randomly assessed the quality of interviews through a quality control data management system. On average, the survey was collected in 18 min using Computer-Assisted Telephone Interviewing software. On June, the largest survey, a total of 53 852 phone calls were effectively dialed, but 2400 numbers were inactive and 34 080 calls sent automatically to voicemail. This volume of invalid numbers is expected in Random Digit Dialing surveys. In 17 374 calls, a conversation ensued, 87 % rejected the interview, 1·2 % were ineligible (i.e. minors), 2 % did not complete the interview and 9·6 % provided a full interview (a reasonable response in Random Digit Dialing surveys). Moreover, the quality assurance system required that all interviewers rate each interview at the end of the call: the majority (96–98 %) considered that the interviewees were interested in responding to the survey and between 94 and 97 % believed that most of the responses to their questions were reliable. Importantly, they reported that they felt the interviewees understood the questions in the survey (94–95 %).

### Measures

The study used three cross-sectional ENCOVID-19 waves (i.e. April, May and June). Household food insecurity was measured with the eight-item adult version of the ELCSA^(22)^, which is the basis of the Food Insecurity Experience Scale^(23)^. ELCSA has been extensively validated for Mexico^(29)^ and is widely used in the country to measure multidimensional poverty^(30)^. It is also included in the Mexican National Health and Nutrition Survey (ENSANUT for its acronym in Spanish). Hence, the last ENSANUT conducted in 2018 was used to compare household food insecurity prevalence before the COVID-19 pandemic.

The ELCSA enquires if, in the last 3 months, due to a lack of money or other resources, the respondent or any other adult in the household: (i) worried you might run out of food (*worried*); (ii) were unable to eat healthy, balanced and nutritious food (*healthy*); (iii) ate only a few kinds of foods (*fewfoods*); (iv) skipped breakfast, lunch or dinner (*skipped*); (v) ate less than s/he thought should have (*ateless*); (vi) ran out of food (*ranout*); (vii) were hungry but did not eat (*hungry*) and (viii) went without eating for a whole day (*whlday*). Responses to all items are dichotomous (i.e. Yes/No). After computing the total summative score for the eight items, food (in)security was categorised into four levels: food security (total score = 0), mild food insecurity (1–3), moderate food insecurity (4–6) and severe food insecurity (7–8). This method of estimation was followed in the current analysis; missing values for the food insecurity questions were minimal, with a maximum of 1·7 % in April.

The usual way to use the ELCSA scale is by repeating, for each item, the 3-month time frame and emphasising lack of money or other resources as the cause to endorse the item. Since telephone surveys need to be short, an adapted version of the ELCSA was used in which the time and lack of resources framing was mentioned only once, before asking the items (see online supplementary material, Supplemental Materials S.1). Interviewers were instructed to repeat it whenever the respondent hesitated on the meaning of an item. The eight-item ELCSA was on average collected in 4 minutes.

Household SES was measured with the assets-based AMAI index^(31)^. It combines six household indicators from the National Income and Expenditure Survey^(2)^: (i) education level of the head of household; (ii) number of complete bathrooms; (iv) number of cars or vans; (v) having Internet connection; (vi) number of household members 14 years or older who are working and (vii) number of bedrooms. Based on a summative score and standard cut-off points, SES is categorised into seven mutually exclusive categories, ranging from ‘A/B’ to ‘E’, where E represents the lowest SES level.

Anxiety was measured with the two-item Generalised Anxiety Disorder scale^(32)^ that inquires about the frequency by which the respondent felt during the last 2 weeks: (i) nervous, anxious or on edge and (ii) not being able to stop or control worrying. Response options are ‘never’; ‘several days’; ‘more than half of days’ and ‘almost every day’. An additive score of the responses was computed (range 0 to 6), and a cut-off point of 3 or more was used to classify as having anxiety disorder symptoms.

The surveys from May and June included a filter question to identify households with individuals under 18 years; this was unavailable for April. Hence, a dummy variable identifying households with children (< 18) or without children was generated to address differences in household food insecurity prevalence between these two types of families.

### Analysis

Statistical analyses were conducted in four steps. First, general reliability estimates compared how consistently the ELCSA scale psychometrics performed in the different samples. Alpha and omega (with tethrachoric correlations) were estimated and compared; according to accepted standards^(33)^, values above 0·7 indicated adequate reliability and suggested high inter-item correlations.

Second, internal psychometric validity was assessed as recommended with the Rasch measurement model^(34)^. The Rasch model shows the extent to which observable items are consistent with the latent phenomenon, i.e. household food insecurity^(35)^. Estimations were conducted with the one-parameter logistic model using a conditional maximum likelihood approach and sampling weights at the household level from each survey^(23)^. Given the eight dichotomous responses to the ELCSA, the models yield item severity parameters indicating the location of the item on the latent variable. Higher severities correspond to items with higher levels of food insecurity and, therefore, with lower item means. Likewise, item severity was estimated for the total summative score as a way to assess if the items were measuring the whole range of the latent trait.

Item performance was assessed through infit statistics, which show the strength and consistency of the association between an item and the latent trait. The Rasch model assumes all items discriminate equally well and, if the assumption is met, infit statistics equal 1; nonetheless, infit values in the range 0·7–1·3 are considered acceptable^(23)^. When the statistic is between 1·3 and 1·5, items can still be used for measurement but should be closely monitored to establish if there is a systematic bias. Items with infit values above 1·5 should be discarded. Infit values below 0·7 are less worrisome because they indicate that the item’s contribution may be redundant.

To assess the comparability of measures of fit between samples, a ‘Flat’ reliability test was performed. ‘Flat’ reliability measures the proportion of total variation in true severity accounted for by the model^(23)^. A good model fit has a reliability between 0·7 and 0·8. Lastly, conditional independence of items was examined with an inspection of residual correlations, where it is expected they do not exceed 0·3.

All the analyses were conducted for four surveys: ENSANUT 2018 and three cross-sections of the ENCOVID-19 (i.e. May to June). ENSAUT 2018 served as the pre-pandemic reference (*n* 44 509). Likewise, the analyses were conducted for households with and without children in the May to June samples.

The third step in the analysis focused in the concurrent validity assessed by correlating the ELCSA score with SES (Spearman correlation) and with anxiety (Welch two-sample test). These analyses were conducted in a pooled dataset of the 3 months using the raw summative score of food insecurity (range 0–8). We hypothesised that food insecurity would have a negative association with SES, and higher levels of anxiety would be found in food insecure households.

In the fourth step, we estimated the prevalence of household food insecurity and assessed how much it changed over the first 3 months of the lockdown. Estimates from ENCOVID-19 were compared with those from ENSANUT 2018. The analysis was performed for the total sample and stratified by households with and without children.

All analyses were conducted in R software; Rasch models (RM) were ran using the RM.weights package, developed by FAO to conduct the statistical validation of the Food Insecurity Experience Scale^(36)^.

## Results

The adapted version of the ELCSA scale used in ENCOVID-19 repeated cross-sectional surveys was found to be reliable. The alpha coefficient for ENSANUT was 0·90, and the values for ENCOVID-19 – April, May and June – ranged between 0·87 and 0·89. A more stringent measure for dichotomous matrices showed similar results. While ENSANUT had an omega coefficient of 0·80, the values of the ENCOVID were between 0·75 and 0·77. Inter-item correlations were above the cut-off point of 0·6 in all surveys.

ELCSA’s item severity parameters in the four surveys followed a similar order as the Food Insecurity Experience Scale global standard^(23)^ (Table 1). Severity parameters inversely mirror the mean’s order and showed how the adapted version of the ELCSA in the ENCOVID-19 was able to measure the full range of the latent trait. ELCSA item’s psychometric performance was adequate in the ENCOVID-19 surveys (Table 1). The only item with an infit statistic above the 1·3 threshold was ‘ran out of food’ (*ranout)*, in May’s wave, with a value of 1·35. This was not a concern since it represented a small deviation from the range, it had the highest infit value in the ENSANUT survey (1·24) and the ‘misfit’ was not systematic. No item had systematic deviations in the outfit values (Table 1).

**Table 1:** Comparison of weighted means and item severity parameters, infit and outfit statistics of the Latin American and Caribbean Food Security Scale (ELCSA) scale between the Mexican National Health and Nutrition Survey (ENSANUT) 2018 and the three ENCOVID-19 surveys from April, May and June 2020

	ENSANUT 2018					ENCOVID-19 April				
Items	Means	Item sev.	SE	Infit	Outfit	Means	Item sev.	SE	Infit	Outfit
Worried	49·3	–3·311	0·022	1·197	2·907	52·8	–2·717	0·139	1·176	3·118
Fewfoods	37	–1·801	0·019	0·846	1·152	36·4	–1·16	0·126	0·883	1·048
Healthy	34·6	–1·491	0·019	0·933	1·325	31·7	–0·667	0·127	0·966	0·899
Ateless	23·5	–0·018	0·02	0·826	0·739	29·4	–0·381	0·128	0·795	0·632
Ranout	15	1·358	0·022	1·235	1·324	18	1·015	0·144	1·023	0·955
Skipped	14·6	1·44	0·023	0·845	0·769	19·2	0·835	0·14	1·001	0·953
Hungry	14·1	1·527	0·023	0·818	0·664	16·7	1·198	0·147	0·869	0·883
Whlday	10·7	2·296	0·026	0·973	0·959	12·3	1·877	0·164	1·051	1·474

	ENCOVID-19 May					ENCOVID-19 June				
Items	Means	Item sev.	SE	Infit	Outfit	Means	Item sev.	SE	Infit	Outfit
Worried	58·3	–2·613	0·139	1·154	1·933	57·7	–2·19	0·087	1·14	1·375
Few foods	31·2	–0·285	0·124	0·922	0·861	38·5	–0·644	0·08	1·062	1·278
Healthy	34·4	–0·607	0·122	1·062	1·3	37·7	–0·589	0·08	1·008	1·028
Ateless	27·7	0·08	0·128	0·863	0·754	31·3	0·01	0·083	0·876	0·825
Ranout	23	0·632	0·136	1·348	1·162	19·5	1·271	0·095	1·049	0·92
Skipped	19·6	1·076	0·144	0·761	0·6	22·9	0·873	0·09	0·84	0·697
Hungry	17·9	1·325	0·149	0·775	0·567	21·3	1·055	0·092	0·868	0·65
Whlday	14·8	1·829	0·162	0·881	0·708	15·4	1·864	0·105	1·081	1·321

sev, severity; Whlday, whole day; all means and Rasch models estimated with household-level sampling weights.

The severity parameters of the raw summative score were equivalent between surveys (Table 2). These results indicate that the summative score of ELCSA from ENCOVID-19 was able to measure equally well the three levels of food insecurity when compared with ENSANUT 2018. Overall model fit was excellent in the ENCOVID-19 surveys. Flat reliability had a value of 0·78 in the ENSANUT survey and was very similar in ENCOVID-19, April (0·74), May (0·73) and June (0·73). The assumption of conditional independence held for most pairs of items. The internal validity in ENCOVID-19 in households with and without children in May and June also confirmed its adequate psychometric performance (see online supplementary material, Supplemental Materials S.2).

**Table 2:** Comparison of the severity parameters from the raw summative score of the Latin American and Caribbean Food Security Scale (ELCSA) scale between the Mexican National Health and Nutrition Survey (ENSANUT) 2018 and the three ENCOVID-19 surveys from April, May and June 2020

Score	ENSANUT 2018		ENCOVID-19 April		ENCOVID-19 May		ENCOVID-19 June	
	Severity	SE	Severity	SE	Severity	SE	Severity	SE
0	–4·106	1·601	–3·589	1·577	–3·363	1·601	–3·187	1·552
1	–3·076	1·252	–2·654	1·218	–2·386	1·239	–2·273	1·180
2	–1·779	1·058	–1·500	0·973	–1·213	0·966	–1·209	0·941
3	–0·875	1·001	–0·656	0·877	–0·399	0·850	–0·429	0·848
4	0·178	0·942	0·080	0·842	0·275	0·809	0·254	0·814
5	1·026	0·907	0·788	0·846	0·933	0·817	0·936	0·823
6	1·815	0·935	1·544	0·908	1·648	0·887	1·631	0·888
7	2·889	1·144	2·539	1·132	2·613	1·120	2·624	1·123
8	3·736	1·601	3·368	1·577	3·429	1·601	3·437	1·552

Rasch models estimated with household-level sampling weights.

**Fig. 1:**
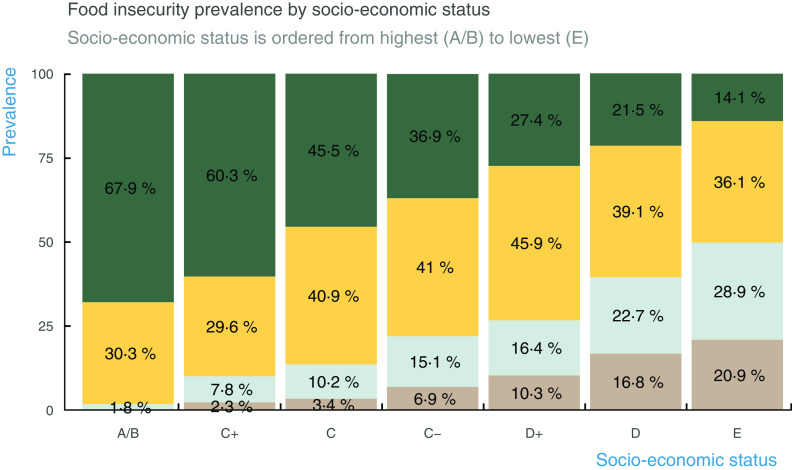
Food insecurity prevalence by socioeconomic status. The graph illustrates the inverse relationship between socio-economic status and food security. Households in the lowest SES level have the highest prevalence of moderate and severe food insecurity (28·9 % and 20·9 %, respectively). Conversely, households in the highest SES level have the lowest prevalence of moderate and severe food insecurity (1·8 % and 0 %, respectively). 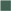, Food security; 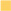, mild food insecurity; 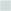, moderate food insecurity; 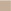, severe food insecurity

Concurrent validity of food insecurity was established by its association with SES and anxiety. The Spearman correlation between the raw score of food insecurity and SES was negative and statistically significant (–0·4; CI: –0·37—0·43). Figure 1 shows a clear dose–response gradient between SES and food insecurity severity. Even though mild food insecurity was present at every SES level, moderate and severe food insecurity increased as SES dropped. At the lowest SES level, moderate and severe food insecurity reached its highest prevalence, at 28·9 % and 20·9 %, respectively. Notably, middle-SES households reported a prevalence of moderate and severe food insecurity from 10 % in C+ to 26 % in D+.

**Fig. 2:**
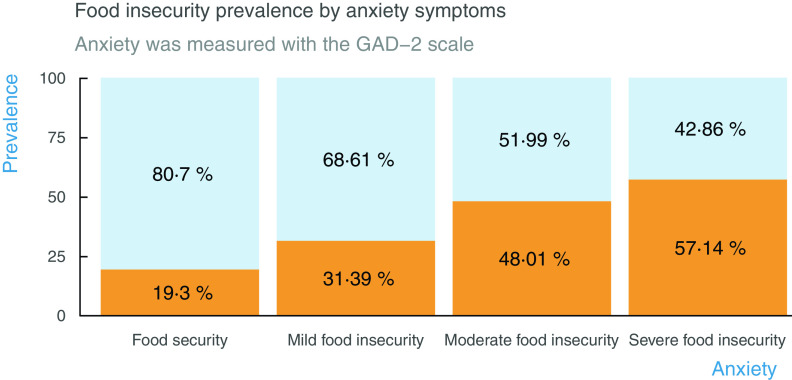
Food insecurity prevalence by anxiety symptoms. The graph illustrates the positive relationship between socio-economic status and anxiety. Persons living in food secure households report fewer anxiety symptoms than persons living in food insecure households. 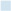, No symptoms; 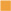, symptoms

Anxiety was also associated with food insecurity in the expected direction. Individuals without anxiety symptoms reported a mean total food insecurity score of 1·68, and this score was substantially higher among individuals with anxiety symptoms (3·11). Figure 2 shows the dose–response gradient between food insecurity and anxiety. While 19·3 % of persons living in food secure households reported symptoms of anxiety, this was the case for 57·1 % among those living in households with severe food insecurity.

**Fig. 3:**
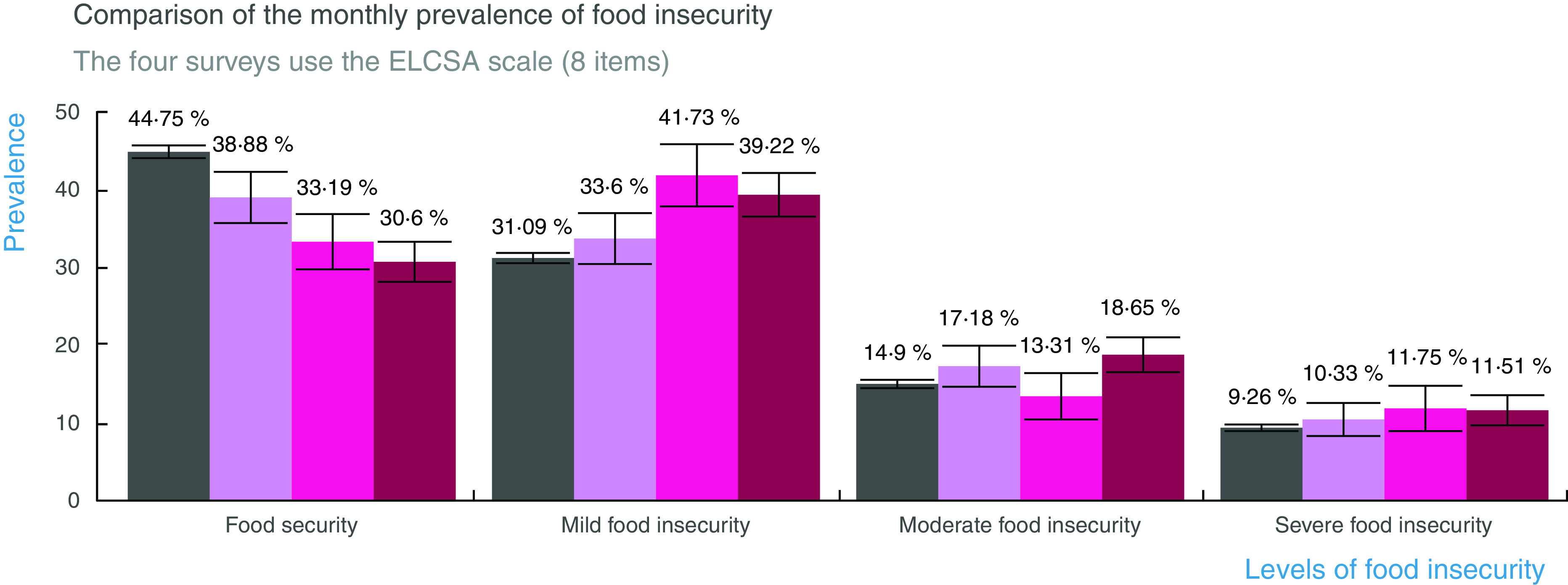
Monthly comparison of the prevalence of food insecurity. In contrast with ENSANUT from the year 2018, food security is decreasing during the COVID-19 pandemic. Mild food insecurity reached its highest level in May, and moderate food insecurity was highest in June. 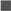, ENSANUT 2018; 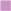, ENCOVID April 2020; 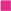, ENCOVID May 2020; 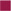, ENCOVID June 2020

Prevalence estimates indicated that the prevalence of food security has been decreasing as the COVID-19 pandemic advances in Mexico (Table 3). According to ENSANUT 2018, 44·7 % of households were food secure, but then it significantly dropped to 38·8 % in April and then to 33·2 % in May and 30·6 % in June 2020 (Fig. 3). Mild food insecurity reached its highest level in May (41·7 %) and moderate food insecurity in June 2020 (18·65 %). Severe food insecurity in June was not statistically different from the 2018 prevalence, very likely as a result of low statistical power; however, the secular trend since the outbreak of the pandemic suggests that severe food insecurity may also be worsening on a monthly basis. This relationship will continue to be monitored as more rounds of ENCOVID-19 data accumulate.

**Table 3:** Prevalence comparisons between the Mexican National Health and Nutrition Survey (ENSANUT) 2018 and the three ENCOVID-19 surveys from April, May and June 2020

	ENSANUT 2018			ENCOVID-19 April			ENCOVID-19 May			ENCOVID-19 June		
	Prop	CI		Prop	CI		Prop	CI		Prop	CI	
Food security	44·75	43·93	45·57	38·88	35·59	42·18	33·19	29·64	36·74	30·6	28·02	33·18
Mild FI	31·09	30·43	31·75	33·6	30·33	36·87	41·73	37·73	45·72	39·22	36·41	42·03
Moderate FI	14·9	14·36	15·43	17·18	14·53	19·82	13·31	10·33	16·29	18·65	16·42	20·89
Severe FI	9·26	8·84	9·68	10·33	8·2	12·45	11·75	8·84	14·66	11·51	9·57	13·45

Prop, proportion; all proportions were estimated with household-level sampling weights. FI, food insecurity.

**Fig. 4:**
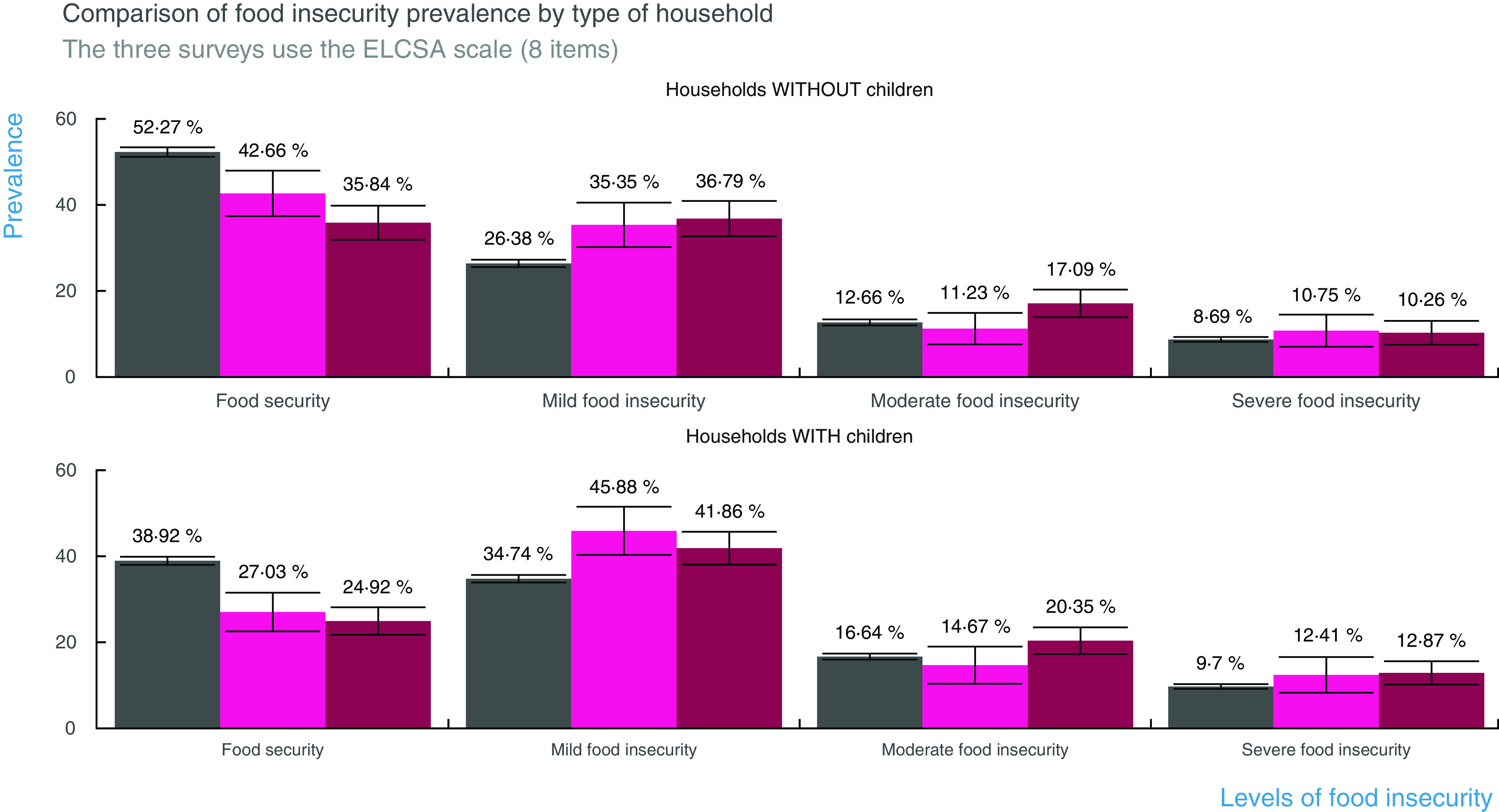
Comparison of the prevalence of food insecurity by type of household. Households with children have lower levels of food security than households without children. Households with children had the highest prevalence of mild food insecurity in May and of moderate food insecurity in June 2020. 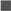, ENSANUT 2018; 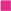, ENCOVID May 2020; 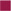, ENCOVID June 2020

The stratified analysis showed that food insecurity was higher in households with than without children (Fig. 4). Food security in households with children decreased from 38·9 % in 2008 to 27 % in May and to 24·9 % in June. The highest prevalence of mild food insecurity was during May (45·8 %) and for moderate food insecurity during June (20·3 %). Severe food insecurity seems to be increasing as well but confidence intervals overlapped with those of ENSANUT 2018 (see online supplementary material, Supplementary Material S.2).

## Discussion

Our findings strongly suggest that the COVID-19 pandemic is increasing food insecurity in Mexico on a monthly basis during the mandated lockdown. These results are consistent with previous studies that demonstrate how in vulnerable households food security declines during socio-economic shocks^(4,5)^ and that as the pandemic advances food security can worsen even further^(7,8)^. Moreover, lockdowns associated with the pandemic might keep disrupting the food supply chain and food consumption patterns. It is especially worrisome that these disruptions might increase all forms of malnutrition during the COVID-19 pandemic, including an additional risk of obesity, due to the consumption of ultra-processed foods and increased sedentarism^(3)^. It is thus necessary to monitor the indirect health consequences of the COVID-19 pandemic.

The adapted version of the ELCSA scale used in the ENCOVID-19 proved to be reliable and valid. Rasch models showed that the high reliability of the telephone version of the scale was comparable with the face-to-face application in ENSANUT 2018. Notably, the scale’s internal consistency was strong on three independent monthly samples, which indicates that the scale was equally reliable and – combined with the Rasch analyses – showed that it can be used with a high degree of confidence to track changes in food insecurity prevalence over time. A limitation of ENCOVID-19, however, was the insufficient inclusion of persons living in rural and isolated localities due to lower mobile phone coverage. Since these communities have higher extreme poverty levels than urban enclaves in Mexico^(37)^, lower coverage of these areas may have led to an underestimation of food insecurity. In spite of these limitations, thanks to ENCOVID-19, Mexico has been able to rapidly and efficiently monitor changes in food security in the general population and in diverse vulnerable locations. The positive results on the validity and reliability of the telephonic ELCSA for tracking food insecurity, together with the strong media coverage and endorsement from government officials of ENCOVID-19 findings, indicate that the main goal of this project is being met. We are now in the process of understanding if and how local and state governments, universities and international and civil society organisations are using the findings from ENCOVID-19 to design strategies and policies to protect the food security and well-being of families during the pandemic and its aftermath and on tracking their impacts on household food security.

Timely and high-quality food insecurity surveillance systems can help governments respond to the major food security challenge posed by the COVID-19 pandemic. Telephone surveys are a feasible and cost-effective strategy to measure food insecurity with experience-based scales such as ELCSA. These results can inform similar strategies in countries using the Food Insecurity Experience Scale, but they might be especially useful for countries in Latin America and the Caribbean that regularly measure food insecurity with the ELCSA scale. ENCOVID-19 is an efficient strategy to regularly monitor changes in several indicators, including food insecurity, and thus can serve as a high-quality instrument that can be frequently applied to inform decision-makers and assess the performance of social protection programmes during public health emergencies and other pressing circumstances^(38)^.

The validity of ELCSA was also examined in its association with other variables. The selected measure of SES is a reliable assets-based scale suitable for implementation in short telephone surveys. It has previously been proved – in face-to-face interviews – to be highly associated with income deciles in all states of Mexico and across localities with different population sizes^(31)^. However, a limitation is that this SES scale is unable to capture changes in economic circumstances, and it only reflects pre-pandemic SES. Nonetheless, households with low SES reported a considerably higher prevalence of food insecurity than those with high SES. Remarkably, food insecurity was reported in 10–26 % of households in the middle of the SES scale – households that may have become newly food insecure as a result of the pandemic. This means that the pandemic is negatively affecting food security in households along the SES continuum, albeit more severely among those with lower SES.

Food insecurity was also associated with anxiety. The pervasive link between food insecurity and stress helped to anticipate these results^(15,39)^, but the pandemic is intensifying it. During the lockdown, individuals living under conditions of moderate and severe food insecurity had more than double the anxiety levels than food secure households. These estimates were similar to what was found in a sample of US households, where anxiety was 2·09 more likely in the presence of food insecurity^(16)^. These results confirm the expected psycho-emotional toll and the complex syndemic interplay of mental health and the experience of food insecurity during the pandemic. As a case in point, it is possible that the increase in food insecurity in households with young children may be leading to more family chaos and poor interpersonal relations^(40)^. Likewise, the stress levels in households with food insecurity may have increased the odds of suffering emotional and physical intimate partner violence^(41)^. It is important to keep monitoring the interaction of the two indicators because these consequences can deepen with the additional stress of the economic and health crisis. Examining these associations over time can indeed shed light on the syndemic effects of food insecurity and mental health on household dynamics during the pandemic^(3)^ and may help understand ways of intervining to address the reinforcing association between anxiety and food insecurity.

Food insecurity is more prevalent in households with children. These households require focalised social protection actions. The definition of children in ENCOVID-19 is not as granular as would have been desirable, as clusters of infants, children and adolescents, which require different policy strategies. Prior studies have already highlighted the vulnerability of young children and their families during the COVID-19 pandemic^(3)^. The findings of the worsening trend of food insecurity stress the urgent need for policy actions. It is important to consider providing and evaluating food assistance and cash transfers, at least to the most vulnerable households. For instance, prior studies have found that a cash transfer equivalent to one minimum wage in Mexico ($176 USD a month) and a waiver of payment of basic services (i.e. electricity, water, etc.) to households with informal jobs and with children under 5 years of age may help protect their food security, and it would require using <0·06 % of Mexico’s GDP^(42)^. Moreover, it is important to establish or expand food assistance programmes including cash transfers for food, food banks and school meals programmes directed to families with children, adolescents, pregnant and lactating women^(3)^. Beyond specific programmes, tackling these convergent syndemic-like crises will require multi-level and evidence-based policies based on a complex adaptive systems framework^(2)^. Even if the COVID-19 recedes, its socio-economic and health effects are expected to last for a long time.

## Conclusion

Household food insecurity is worsening as the COVID-19 pandemic advances in Mexico. The causal web between the socio-economic shocks, food insecurity and adverse health and mental health outcomes needs to be addressed as a complex syndemic with comprehensive and multi-level policy actions. The results of the study show that the ELCSA scale is a valid and reliable instrument to track food insecurity in the general population and among key vulnerable groups, such as those with low and middle SES. Governments should not attempt to navigate the COVID-19 pandemic blindfolded. High-quality and cost-effective strategies to monitor food insecurity are available and should be implemented widely.

## Supplementary material

For supplementary material accompanying this paper visit https://doi.org/10.1017/S1368980020004000. click here to view supplementary material
