# Supplementary material for: Food insecurity measurement and prevalence estimates during the COVID-19 pandemic in a repeated cross-sectional survey in Mexico
Source: Public Health Nutr. 2020 Oct 14:1–10. doi: 10.1017/S1368980020004000 (PMC7653232; doi:10.1017/S1368980020004000)
Supplement: Supplementary file 1 [file S1368980020004000sup001.docx]

**Supplementary Materials**

**S.1** Adapted version of the ELCSA scale included in the ENCOVID-19. Original version in Spanish and translation to English.

En los últimos 3 meses, por falta de dinero u otros recursos, alguna vez…

|  | Sí | No | No Sabe/ No Responde |
| --- | --- | --- | --- |
| 1. ¿Se preocupó de que los alimentos se acabaran en su hogar? |  |  |  |
| 1. ¿Se quedaron sin alimentos en su hogar? |  |  |  |
| 1. ¿Dejaron de tener una alimentación saludable (nutritiva, balanceada y equilibrada)? |  |  |  |
| 1. ¿Usted o algún otro adulto tuvo una alimentación basada en poca variedad de alimentos? |  |  |  |
| 1. ¿Usted o algún otro adulto dejó de desayunar, comer, o cenar? |  |  |  |
| 1. ¿Usted o algún otro adulto comió menos de lo que debía comer? |  |  |  |
| 1. ¿Usted o algún otro adulto sintió hambre, pero no comió? |  |  |  |
| 1. ¿Usted o algún otro adulto comió sólo una vez o dejó de comer todo un día? |  |  |  |

In the last three months, due to a lack of money or other resources, have you ever…

|  | Yes | No | Don’t know/ Does not answer |
| --- | --- | --- | --- |
| a. Worried you might run out of food? |  |  |  |
| b. Ran out of food in your household? |  |  |  |
| c. Were unable to eat healthy, balanced, and nutritious food? |  |  |  |
| d. You or any other adult in the household, ate only a few kinds of foods? |  |  |  |
| e. You or any other adult in the household, skipped breakfast, lunch or dinner? |  |  |  |
| f. You or any other adult in the household, ate less than s/he thought should have? |  |  |  |
| g. You or any other adult in the household, were hungry but did not eat? |  |  |  |
| h. You or any other adult in the household, went without eating for a whole day? |  |  |  |

**S.2.** Rasch models for internal validity of the ELCSA scale and prevalence estimates for households with and without children in May and June 2020 using the ENCOVID-19 surveys.

**S.2a.** Comparison of weighted means and item severity parameters, infit and outfit statistics of the ELCSA scale between the ENSANUT 2018 and two ENCOVID-19 surveys from May and June 2020 by type of household

|  | **ENCOVID-19 May** | | | | | | | | | |
| --- | --- | --- | --- | --- | --- | --- | --- | --- | --- | --- |
|  | Households without children | | | | | Households with children | | | | |
| **Items** | **Means** | **Sev.** | **St.err.** | **Infit** | **Outfit** | **Means** | **Sev.** | **St.err.** | **Infit** | **Outfit** |
| *worried* | 48.8 | -2.574 | 0.194 | 1.111 | 1.828 | 64.5 | -2.781 | 0.195 | 1.178 | 2.027 |
| *fewfoods* | 26.1 | -0.283 | 0.191 | 1.064 | 1.061 | 34.5 | -0.413 | 0.165 | 0.863 | 0.765 |
| *healthy* | 31 | -0.890 | 0.183 | 1.113 | 1.315 | 36.6 | -0.602 | 0.164 | 1.026 | 1.272 |
| *ateless* | 24 | -0.001 | 0.197 | 0.844 | 0.791 | 30.1 | -0.008 | 0.170 | 0.874 | 0.737 |
| *ranout* | 21.1 | 0.424 | 0.206 | 1.202 | 0.930 | 24.2 | 0.603 | 0.182 | 1.422 | 1.297 |
| *skipped* | 15.9 | 1.325 | 0.231 | 0.961 | 0.975 | 22.1 | 0.848 | 0.188 | 0.660 | 0.453 |
| *hungry* | 17.4 | 1.051 | 0.222 | 0.595 | 0.317 | 18.3 | 1.332 | 0.201 | 0.851 | 0.721 |
| *whlday* | 14.1 | 1.695 | 0.246 | 0.846 | 0.998 | 15.2 | 1.771 | 0.216 | 0.899 | 0.529 |
|  | **ENCOVID-19 June** | | | | | | | | | |
|  | Households without children | | | | | Households with children | | | | |
| **Items** | **Means** | **Sev** | **St.err.** | **Infit** | **Outfit** | **Means** | **Sev.** | **St.err.** | **Infit** | **Outfit** |
| *worried* | 49.9 | -1.921 | 0.125 | 1.196 | 1.365 | 66.2 | -2.736 | 0.130 | 1.079 | 1.329 |
| *fewfoods* | 38.1 | -0.952 | 0.120 | 0.988 | 1.263 | 39 | -0.555 | 0.109 | 1.132 | 1.316 |
| *healthy* | 36.5 | -0.835 | 0.121 | 0.965 | 1.039 | 39 | -0.564 | 0.109 | 1.056 | 1.025 |
| *ateless* | 29.7 | -0.168 | 0.125 | 0.972 | 0.986 | 33.1 | -0.032 | 0.111 | 0.785 | 0.684 |
| *ranout* | 17 | 1.300 | 0.149 | 0.913 | 0.810 | 22.3 | 1.046 | 0.123 | 1.177 | 1.000 |
| *skipped* | 20.7 | 0.819 | 0.139 | 0.872 | 0.633 | 25.2 | 0.719 | 0.118 | 0.818 | 0.833 |
| *hungry* | 19.2 | 1.006 | 0.143 | 0.923 | 0.738 | 23.6 | 0.898 | 0.121 | 0.821 | 0.564 |
| *whlday* | 14.8 | 1.628 | 0.158 | 1.042 | 1.519 | 16 | 1.892 | 0.143 | 1.118 | 0.925 |

NOTE: sev, severity; St.err, Standard Error. All means and Rasch models estimated with household level sampling weights.

**S.2b.** Comparison of the severity parameters from the raw summative score of the ELCSA scale between the ENSANUT 2018 and two ENCOVID-19 surveys from May and June 2020 by type of household

|  | May | | | | June | | | |
| --- | --- | --- | --- | --- | --- | --- | --- | --- |
|  | Without children | | With children | | Without children | | With children | |
| **Raw-Score** | **Severity** | **St.err** | **Severity** | **St.err** | **Severity** | **St.err** | **Severity** | **St.err** |
| 0 | -3.405 | 1.583 | -3.487 | 1.612 | -3.192 | 1.521 | -3.474 | 1.604 |
| 1 | -2.462 | 1.221 | -2.498 | 1.254 | -2.336 | 1.150 | -2.499 | 1.244 |
| 2 | -1.310 | 0.959 | -1.294 | 0.970 | -1.312 | 0.922 | -1.319 | 0.968 |
| 3 | -0.503 | 0.852 | -0.493 | 0.853 | -0.542 | 0.844 | -0.504 | 0.852 |
| 4 | 0.183 | 0.812 | 0.198 | 0.809 | 0.145 | 0.818 | 0.179 | 0.810 |
| 5 | 0.841 | 0.820 | 0.854 | 0.818 | 0.816 | 0.827 | 0.836 | 0.819 |
| 6 | 1.561 | 0.890 | 1.570 | 0.889 | 1.546 | 0.893 | 1.555 | 0.891 |
| 7 | 2.525 | 1.120 | 2.536 | 1.121 | 2.519 | 1.122 | 2.528 | 1.125 |
| 8 | 3.340 | 1.583 | 3.353 | 1.612 | 3.326 | 1.521 | 3.351 | 1.604 |

NOTE: St.err, Standard Error. Rasch models estimated with household level sampling weights.

**S3c.** Prevalence comparisons between the ENSANUT 2018 and two ENCOVID-19 surveys from May and June 2020 by type of household

| Households without children | | | | | | | | | |
| --- | --- | --- | --- | --- | --- | --- | --- | --- | --- |
|  | **ENSANUT 2018** | | | **May** | | | **June** | | |
|  | **Prop** | **IC** | | **Prop** | **IC** | | **Prop** | **IC** | |
| **Food Security** | 52.27 | 51.16 | 53.37 | 42.66 | 37.36 | 47.95 | 35.84 | 31.87 | 39.80 |
| **Mild** | 26.38 | 25.53 | 27.25 | 35.35 | 30.19 | 40.51 | 36.79 | 32.70 | 40.89 |
| **Moderate** | 12.66 | 11.97 | 13.38 | 11.23 | 7.56 | 14.89 | 17.09 | 13.88 | 20.30 |
| **Severe** | 8.69 | 8.14 | 9.28 | 10.75 | 7.01 | 14.48 | 10.26 | 7.48 | 13.05 |
| Households with children | | | | | | | | | |
|  | **ENSANUT 2018** | | | **May** | | | **June** | | |
|  | **Prop** | **IC** | | **Prop** | **IC** | | **Prop** | **IC** | |
| **Food Security** | 38.92 | 37.97 | 39.87 | 27.03 | 22.52 | 31.53 | 24.92 | 21.73 | 28.12 |
| **Mild** | 34.74 | 33.87 | 35.62 | 45.88 | 40.29 | 51.48 | 41.86 | 38.04 | 45.67 |
| **Moderate** | 16.64 | 15.96 | 17.34 | 14.67 | 10.35 | 18.98 | 20.35 | 17.23 | 23.46 |
| **Severe** | 9.7 | 9.16 | 10.25 | 12.41 | 8.26 | 16.55 | 12.87 | 10.15 | 15.58 |

NOTE: All proportions were estimated with household level sampling weights. Prop, Proportion; CI, Confidence interval.
